# Enhanced Transmissions Through Three-dimensional Cascade Sharp Waveguide Bends Using C-slit Diaphragms

**DOI:** 10.1038/srep45095

**Published:** 2017-03-21

**Authors:** Rui Yang, Bowei Hu, Aofang Zhang, Dongxing Gao, Hui Wang, Ayuan Shi, Zhenya Lei, Pei Yang

**Affiliations:** 1 School of Electronic Engineering. Xidian University, Xi’an 710071, People’s R China

## Abstract

Transmission properties through sharp rectangular waveguide bends are investigated to determine the cut-off bending angles of the wave propagation. We show that a simple metallic diaphragm at the bending corner with properly devised sub-wavelength defect apertures of C-slits would be readily to turn on the transmissions with scarce reflections of the propagating modes, while preserving the integrity of the transmitting fields soon after the bends. In particularly, our design also demonstrates the capability of eliminating all the unwanted cavity resonant transmissions that exist in the three-dimensional cascade sharp waveguide bends, and solely let the desired signals travel along the whole passage of the waveguide. The present approach, using C-slit diaphragms to support the sharp bending behaviors of the guided waves with greatly enhanced transmissions, would be especially effective in constructing novel waveguides and pave the way for the development of more compact and miniaturized electromagnetic systems that exploit these waveguide bends.

Bending light in the free space has been intensively studied nowadays as a result of their great potentials for various advanced applications, such as invisible cloaks^1^,^2^. In the meanwhile, controlling guided modes has also attracted large-scale attentions due to the increasingly quest for the more compact and miniaturized electromagnetic systems. Especially, waveguide bends, functioning as the basic components in the microwave and optical integrated circuits, have therefore always been a focus of the research activities that are exploring new potential configurations with sharper bending angles for the high packing density while possessing the merits of scarce reflections for the transmitting signals^3^,^4^,^5^,^6^,^7^,^8^,^9^,^10^,^11^,^12^,^13^,^14^,^15^,^16^,^17^,^18^,^19^,^20^,^21^,^22^,^23^,^24^,^25^,^26^,^27^.

Traditional strategies to build-up waveguide bends normally require long transit route to redirect the guided modes with gradually bending behaviors to avoid additional losses, or alternatively, utilize the corner mirror or insert higher/lower refractive-index dielectrics at the bending corner to make the guided modes go in the directions that are different from the original way with the sharper bended transmissions for only limited angles^3^,^4^,^5^,^6^,^7^,^8^. Contemporary technologies, on the other hand, are demonstrated being capable of creating desired bending of the guided modes by filling the waveguides with meta-materials, using photonic crystals, or prescribing specific metallic chain particles on the surfaces. For instance, one may use gradient meta-materials to lead guided fields^9^,^10^,^11^,^12^,^13^,^14^,^15^, or alternatively, employ epsilon-near-zero meta-materials to achieve tunneling effects to guide the electromagnetic fields in more arbitrary ways^16^,^17^,^18^,^19^,^20^. In addition, photonic crystals are very competent in guiding and bending the electromagnetic fields, where perfect transmissions through sharp bends could be achieved by adjusting the length of the bend section^21^,^22^,^23^. Especially, surface plasmon polaritons (SPPs) as a kind of quasi two-dimensional (2D) travelling modes sustaining at the interface between metals and air are also shown to be smoothly bended either by the transformation optics derived passages^24^,^25^,^26^ or the ultrathin metallic films as a spoof SPPs travelling channel^27^. These modern technologies have greatly strengthened the controlling ability of the guided waves in many different scenarios compared with the conventional methods and provided promising applications for the compact electromagnetic circuits. However, the practical implementation of the waveguide bends with meta-material fillers is still challenging as all the metamaterial properties are fundamentally depending on the periodic compositions. Especially when it comes to the advanced electromagnetic systems that require miniaturized and multiple-scale bended waveguide components, the operation through filling meta-materials in the bending corner would become increasingly difficulty. In addition, the present researches to create waveguide bends with high transmissions mainly focus on the 2D controlling of the guided modes for the wave bending behaviors solely within the H-plane or the E-plane, and the cascade waveguide structures concerning the three-dimensional (3D) manipulation of the transmitting fields are scarcely mentioned. Especially, the cascade connection of the waveguide often suffers the drawbacks of unwanted cavity resonant transmissions. Therefore, the pursuit of efficient waveguide bend design is still expected, especially for those possessing the simple and compact structures for the practical engineering, but also own the abilities of controlling the guided modes at will to accomplish various scenarios in particularly for the 3D applications.

With these considerations, we propose to open the extraordinary transmission (ET) regime through the sharp waveguide bends using C-slit diaphragms^28^,^29^. Different from the ETs applied to the periodic arrays of subwavelength apertures that are significantly smaller than half-wavelength^30^,^31^,^32^,^33^, our strategy is based on the magnetic resonance induced high transmissions through the single or multiple subwavelength apertures in the metal screen, which is the complementary method as the split-ring-resonator-coupled enhanced transmissions with electrical resonances^34^,^35^. We show that such a simple metallic diaphragm at the bending corner with properly devised sub-wavelength defect apertures of the C-slits would be readily to turn on the transmissions of the propagating modes for both the H-bend and the E-bend with scarce reflections and also preserve the integrity of the transmitting fields soon after the bends. In particularly, such a C-slit diaphragm at the bending corner has the merits of tunable transmission bandwidth to even very wide frequency ranges rather than the inherently narrow band transmission by employing the periodic topology of the subwavelength aperture array to achieve ET peaks, and the transmission frequencies can also be easy to manipulate by simply tuning the C-slit perimeter. Both of the tunable transmission bandwidth and controllable transmission frequency of such C-slits at the bend corner offer us promising potentials for proposing novel waveguide components, especially for the design to eliminate all the unwanted cavity resonant transmissions that exist in the 3D cascade sharp waveguide bends, and solely let the desired signals travel along the whole passage of the waveguide. Our strategy, using the C-slit diaphragms to support the sharp bending behaviors of the guided waves with greatly enhance transmissions, would be especially effective in constructing novel waveguides and pave the way for the development of more compact and miniaturized electromagnetic systems that exploit these waveguide bends.

Let us start with the 2D sharp rectangular waveguide bends only in the H-plane or the E-plane, as shown in Fig. 1(a and (b)). The available bending angle *θ*_*H*/*E*_ relies on the waveguide length *l* of each segment with the relationship of 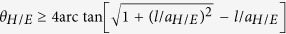 where *a*_*H*/*E*_ is the length of the folded wall of the rectangular waveguide referring to the wide side *AB* for the H-bend or the narrow side *CD* for the E-bend. For example, the minimum available bending angle would reach 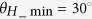 and 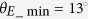 if the waveguide segment has *l* = 90 mm in the WR90 waveguide bends with *a*_*H*_ = 22.86 mm and *a*_*E*_ = 10.16 mm. Wave propagation in such waveguide bends greatly depends on bending angles, and we can clearly observe in Fig. 1(c) that the transmission hits the lowest values at *θ*_*H*_ = 64° and *θ*_*E*_ = 59° for the H-bend and the E-bend respectively at 10 GHz as the first cut-off bending angle (BA) from the straight waveguide. Such a cut-off BA increases when the working frequency moves higher in the X-band, and we can also observe in Fig. 1(d) that H-bend always have a 5-degree larger cut-off BA compared with the E-bend at 10 GHz. As a result, either the H-bend with *θ*_*H*_ = 64° or the E-bend with *θ*_*E*_ = 59° should have scarce energy going across the bending corner around 10 GHz although there is no blockage existing there, as shown in Fig. 1(e) and (f). The minimum transmission for the 64° H-bend happens at 9.96 GHz, while the 59° E-bend is shown to have the lowest transmission at 9.99 GHz within the X-band.

We expect to reboot the stopped energy of the propagating mode of TE_10_ by creating the high transmission waveguide bends while maintaining the field integrity after the bends. In addition, we require using simple structural designs for the practical implementations to construct sharp waveguide bends possessing short transit route for various applications. To this end, the diaphragm consisting of solely a metallic sheet with a C-slit in its center is employed, where four statuses of the C-slit are examined to characterize the overall transmission property of the waveguide bends. We define the 1st-status of the C-slit in the diaphragm having the orientation as shown in Fig. 2(a) and (b) with the split side being parallel to the folded wall *AB* and *CD* of the rectangular waveguide. After an anticlockwise rotation of 90 degrees, we can have the 2nd-status of the C-slit with the split side perpendicular to the folded wall of the rectangular waveguide, while being in the direction of the bending corner. In this way, the 3rd-status of the C-slit should have the same functionality as the 1st-status due to the symmetrical architecture of the waveguide bends, and the 4th-status of the C-slit would thus make the split side perpendicular to the folded wall of the rectangular waveguide, but in the reverse direction of the bending corner. Figure 2(c) to (f) demonstrate the transmission spectrums of the H-bend and the E-bend respectively with the diaphragm having the C-slit at different four statuses. We can observe that the diaphragm with the C-slit at the 1st-status or the 3rd-status in Fig. 2(c) would enable the electromagnetic fields to go penetrating the H-bend and the E-bend at the devised frequency with band-pass responses. The released frequency could be simply tuned through the C-slit perimeter *c* = *a* + 2*b* − 2*w* ≈ *mλ*_*r*_/2 (*m* = 1, 2, 3…) by keeping other structural parameters unchanged, where *a* and *b* refer to the side length of the C-slit, *w* refers to the slit width, and *λ*_*r*_ thus refers to the resonant transmission wavelength. Therefore, the 0.5 mm width C-slit at 1st- or the 3rd-status with different side lengths would lead to different transmission frequencies for the waveguide bends as shown in Fig. 2(c). We can also observe that the transmission bandwidth through H-bend is shown to be smaller than that of the E-bend at the same frequency by including the diaphragms with the 1st-status or the 3rd-status of the C-slit. On the other hand, as shown in Fig. 2(d), the C-slit diaphragm based H-bend happens to have the complete reflection and stopband property with the 2nd-status and the 4th-status C-slit, due to the fact that the direction of the E-field component in the propagating field is now absolutely parallel to the split side of the C-slit, although the structural parameters are completely the same as the 1st-status C-slit at 10 GHz. In the meanwhile, the E-bend with these two statuses of the C-slit are still presenting the tunneling transmissions, possessing wideband response from 9.5 GHz to 11.5 GHz for the 2nd-status C-slit case and extremely narrow transmitting band at 10.21 GHz for the 4th-status C-slit case. The width impacts of the C-slits on the transmission properties of the waveguide bends are also considered for the above four statuses in both the H-bend and the E-bend. We can observe in Fig. 2(e) that the transmissions would move up to the higher frequencies as the aperture become larger by increasing the width of the C-silts from original 0.5 mm to 1 mm and 1.5 mm, while maintaining the side length to be the same as 5.34 mm and 5.40 mm for H-bend and E-bend. Such shifts to the higher frequency transmission are reasonable since the perimeter of the C-slits actually becomes smaller when we increase the width of the C-slit and keep the side length unchanged. In the meanwhile, we can also observe in Fig. 2(f) that the H-bend still have the complete stopband with the 2nd-status and the 4th-status C-slit diaphragm regardless of the aperture size. However, the wideband performance of the E-bend is not properly maintained with larger 2nd-status C-slit, while the original very narrow transmissions of the E-bend are still there but have wider bandwidths and higher resonant frequencies when incorporated with the larger width of the 4th-status C-slit.

We now consider the cascade waveguide bends having an H-bend and an E-bend with one segment of the transmitting route shared with each other, as shown in Fig. 3(a). The C-slit diaphragms with three possible combinations of different status C-slits in the H-bend and in the E-bend are implemented to such a cascade structure of the waveguide bends, where the transit waveguide in the middle would thus experience both of the bending behaviors, leading the released fields in the waveguide to go into 3D as a twisted N-shaped channel, as shown in Fig. 3(b). Given the bending angles as *θ*_*H*_ = 64° and *θ*_*E*_ = 59°, the minimum length of the transit waveguide should be 54.5 mm, otherwise the first and the third segment in the twisted N-shaped waveguide bend would be intertwined with each other. Moreover, the transit waveguide would significantly influence the final transmission spectrum of the whole waveguide bends and additional resonant transmissions are emerging as the transit waveguide segment increases. We can observe in Fig. 3(c) that the twisted N-shaped waveguide bends would have up to four different transmissions within the X-band even with no C-slit diaphragm when the transit waveguide segment changes from 80 mm to 120 mm. The transit waveguide here can be considered as an open ended resonant cavity, where the bending corners together with the first and the third waveguide segments thus function as the resonator terminals. The transmitting wave at the resonant frequency would create very strong cavity fields in the middle segment of the cascade waveguide and promotes the overall transmission for such a cascade waveguide bend, although signals at these frequencies have no chance to completely penetrate the isolated single H-bend or the single E-bend. However, the minimum transmission of such cascade waveguide bends all maintain the same frequency at 9.96 GHz regardless of the transit length of the waveguide. We can also observe that from Fig. 3(d) to (f) the C-slit diaphragm based cascade waveguide bends would be able to release the signals around 10 GHz through the twisted N-shaped channel with the C-slit diaphragm induced tunneling. However, it is needed to note that the cavity resonant transmissions from the transit waveguide still exist when both bandpass diaphragms are employed simultaneous having the 1st-stutas C-slit in H-bend together with the 1st-stutas or the 4th-stutas C-slit in the E-bend, although the C-slit diaphragms in each individual the waveguide bend only allow the single passing band in the transmission. On the other hand, we happen to find that the cascade waveguide bend with the 1st-stutas C-slit in the H-bend and the 2nd-stutas C-slit in the E-bend would completely inhibit all other cavity resonant transmissions regardless the length of the transit waveguide segment, while solely letting the C-slit diaphragm devised signal around 10 GHz penetrate the whole passage of the waveguide bend. The simple physics behind is that the 2nd-stutas C-slit in the E-bend with wideband response conceal the cavity resonant behavior of the transit waveguide, making the middle waveguide segment and the terminal waveguide function together as an integrated passing channel for the electromagnetic fields. Therefore, the original cavity resonances within the different transit waveguide are completely eliminated, and the transmitting signals at these frequencies can no longer get through the whole passage of the waveguide bend, leaving the passing frequency of the signals only determined by the 1st-stutas C-slit in the H-bend.

Figure 4 demonstrates the E-field distributions in the twisted N-shaped passage in the waveguide with the start and the end waveguide segment of 90 mm, and the middle waveguide segment of 120 mm. The TE_10_ mode would stop at the first encountered bending corner either when we feed the structure from the H-bend or the E-bend for the given cascade waveguide structure at 10 GHz. Almost all the transmitting fields are reflected back to the feeding port with little energy going into the transit waveguide segment, as shown in Fig. 4(a) and (b). However, when we add the diaphragms in the bending corners with the 1st-status C-slit in the H-bend and the 2nd-status C-slit in the E-bend, the C-slits are shown to be able to turn on the original stopped transmission at 10 GHz as demonstrated in Fig. 4(c) and (d). The 3D cascade waveguide bends with such C-slit diaphragms would enable the TE_10_ mode to experience the same propagation as in the straight waveguide at the first place, go tunneling through the C-slit diaphragm, and continue to travel in the next waveguide after the bending corner. We can also observe that the squeezed TE_10_ mode from the C-slit would get recovered soon after the bending corner. One half wavelength of the propagating mode would always distribute across the wide wall with varied magnitudes at different transverse section, which behaves as the typical form of the TE_10_ mode before and after the bending corner. Therefore, our diaphragm with properly devised C-slit at the bending corner would switch on the transmissions of the 3D cascade waveguide bends with nearly unit transmission while preserving the integrity of the transmitting fields soon after the bends.

In Fig. 5, we demonstrate the implementation of the nested C-slits in the diaphragm for multiple frequency transmissions through such a 3D cascade sharp waveguide bends. Three 1st-status C-slits are devised within a single diaphragm in the H-bend to promote the transmissions at 9 GHz, 10 GHz, and 11 GHz respectively, while one optimized 2nd-status C-slit is employed in the E-bend to have the all-pass performance to cover the entire X-band, as shown in Fig. 5(a) and (b). In this way, the 3D cascade waveguide bend would enable all the energy at 9 GHz, 10 GHz, and 11 GHz to penetrate through the whole passage as we expected, while being capable of inhibiting the unwanted resonance frequencies from the transit waveguide segment. The nested C-slits in diaphragms should be optimized before they can be competent, and our strategy would focus on tuning the perimeters of the C-slits, and keep the 10 GHz C-slit in the middle of the three nested C-slits still locating the center of diaphragm. The optimization of the 2nd-status C-slit in the E-band is implemented through tuning the lengths of transverse and longitudinal sides to obtain the whole band tunneling transmission effect with the C-slit still being placed in the center of the diaphragm. Figure 5(c) presents the multiband and wideband performances respectively in the C-slit diaphragm based isolated single H-bend and single E-bend. We can observe that the H-bend would open itself for the signals at three different frequencies, and the bandwidth would become narrower when the frequency goes up. The final reached 2nd-status C-slit in diaphragm in the E-bend would give the all-pass transmission with scarce degradation in the X-band. Figure 5(d) thus demonstrates the overall performance of the multiband 3D cascade sharp waveguide bends, and we can observe that final transmission spectrum only represents three peaks at 9 GHz, 10 GHz, and 11 GHz as we expected. For the comparison purpose, we also illustrate the performance of 3D cascade sharp waveguide bends with multiband C-slit diaphragm with the three nested 1st-status C-slit in the H-bend as in Fig. 5(b), and the wideband C-slit diaphragm having the original 2nd-status C-slit as in Fig. 2(b). We can observe that the final transmission spectrum in this case would experience small fluctuations around the 9 GHz transmission, and also possess an additional transmission peak around 11.75 GHz from the resonant transmission of the transit waveguide. These unwanted transmission behaviors are due to the fact that the original wideband 2nd-status C-slit cannot perfectly open the E-band, and has degraded transmission below 9.5 GHz and above 11.5 GHz. Clearly, we can greatly improve such a situation by using optimized 2nd-status C-slit in the E-bend as in Fig. 5(a) and (b), and the overall performance of the 3D cascade waveguide bend is show to have the satisfactory performance of multiband transmissions as we expected. Figure 6 continues to demonstrate the E-field distribution through the 3D cascade waveguide bend. We can observe that the TE_10_ mode at different frequencies of 9 GHz, 10 GHz, and 11 GHz would always be capable of going through the bending corners of the twisted N-shaped waveguide regardless we start the excitation from the H-bend or the E-bend. We can also observe that the transmitting fields would penetrate the corresponding C-slits in the H-bend at 9 GHz, 10 GHz, and 11 GHz respectively, which further verify our strategy to control guide modes in the cascade waveguide bend by employing multi-band C-slit diaphragm in H-bend to determine the expected signals, and using wideband C-slit diaphragm in E-bend to eliminate all the resonant transmissions induced by the transit waveguide cavity and simultaneously enable all the transmissions released by the H-bend.

Finally, we fabricate the C-slit diaphragm based the 3D cascade sharp waveguide bends with the series connected H-bend of *θ*_*H*_ = 64° and E-bend of *θ*_*E*_ = 59°, as shown in Fig. 7(a) and (b), to verify the effectiveness of our proposed design. In the experiments, we start with the test of the bare waveguide bends, and the transmission turns out to have four passing bands as the same with simulations in the X-band, as shown in Fig. 7(c). We continue the measurements by inserting the C-slit diaphragms in the waveguide bending corners to see whether such a cascade waveguide bend would turn on the original notched transmission at C-slit diaphragm devised frequencies. In Fig. 7(d), we can observe that the transmission spectrum turns out to have the transmission peak around 10 GHz when we equip the 3D cascade sharp waveguide bends with diaphragms having 1st-stutas C-slit in the H-bend and the 2nd-stutas C-slit in the E-bend. In addition, the 3D cascade sharp waveguide bends also demonstrate the multiple released wavelengths around 9 GHz, 10 GHz, and 11 GHz with the diaphragm having the three nested 1st-stutas C-slits in the H-bend together with the optimized 2nd-stutas C-slit in the E-bend. From these measurement data, we can observe that there is a little degradation for the transmitting energy from the measured results, and such a discrepancy mainly comes from the metallic loss in the fabrication whereas we assume the whole system are perfectly lossless in the simulation. We can also observe that there are small shifts of the transmission frequencies in the experiment results with the maximum frequency shift of 0.13 GHz. This mainly comes from the machining differences of the C-slit sizes during the fabrication and also the 0.3 mm thickness of the C-slit diaphragms in the practical implementation, where in the simulation we assume the diaphragms are zero thickness. However, the overall transmission from the measurements still maintain the same trend as simulations for the devised passbands, thus providing further verifications of our proposed designs.

In conclusion, we have demonstrated the creation of 3D cascade sharp waveguide bends with greatly enhanced transmissions through C-slit diaphragm in this paper. We show our strategy of using diaphragms with properly devised C-slits at the bending corner could be readily to turn on the transmissions with scarce reflections of the propagating modes, while being capable of perfectly preserving the integrity of the transmitting fields soon after the bends. In particularly, our design has also demonstrated the capability of eliminating all the unwanted cavity resonant transmissions that exist in the 3D cascade sharp waveguide bends, and solely let the desired signals travel along the whole passage of the waveguide. We expect our proposed design by using the C-slit diaphragms to support the sharp bending behaviors of the guided waves would function effectively in constructing novel waveguides and pave the way for the development of more compact and miniaturized electromagnetic systems.

## Additional Information

**How to cite this article:** Yang, R. *et al*. Enhanced Transmissions Through Three-dimensional Cascade Sharp Waveguide Bends Using C-slit Diaphragms. *Sci. Rep.* **7**, 45095; doi: 10.1038/srep45095 (2017).

**Publisher's note:** Springer Nature remains neutral with regard to jurisdictional claims in published maps and institutional affiliations.

## Figures and Tables

**Figure 1:**
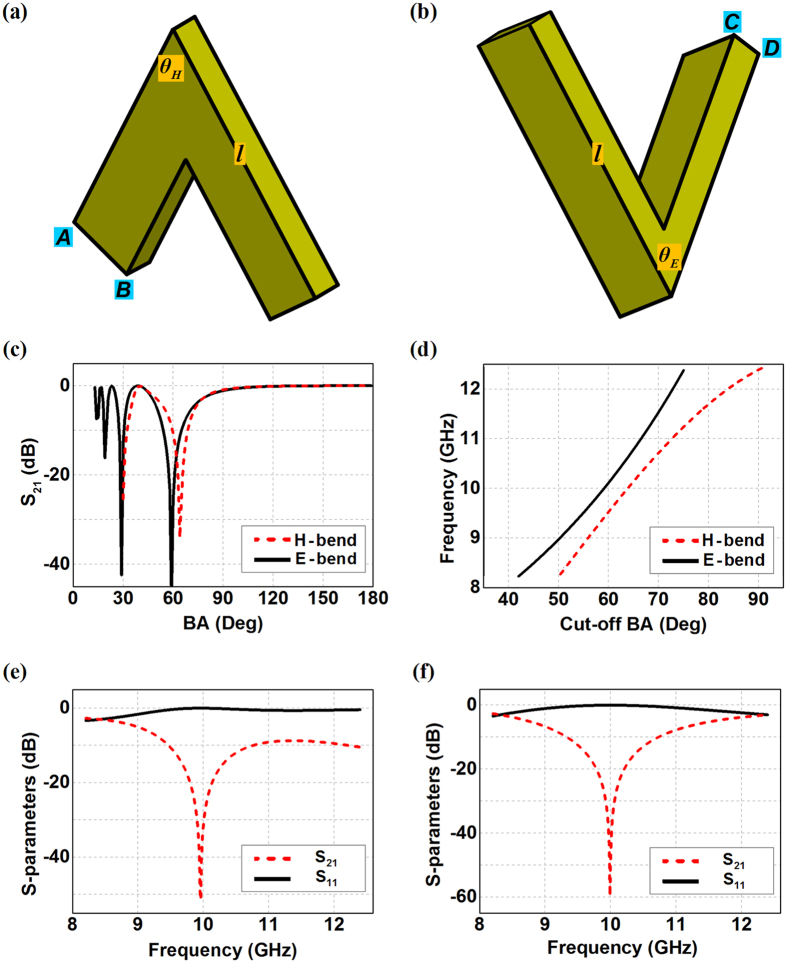
Configurations of the sharp rectangular waveguide bends and their transmission properties. (**a**) H-bend. (**b**) E-bend. (**c**) The overall transmissions of the waveguide bends varied with the bending angle. (**d**) The relationship between the cut-off bending angle and the corresponding frequency. (**e**) The transmission and the reflection of the 64-degree H-bend in the X-band. (**f**) The transmission and the reflection of the 59-degree E-bend in the X-band.

**Figure 2:**
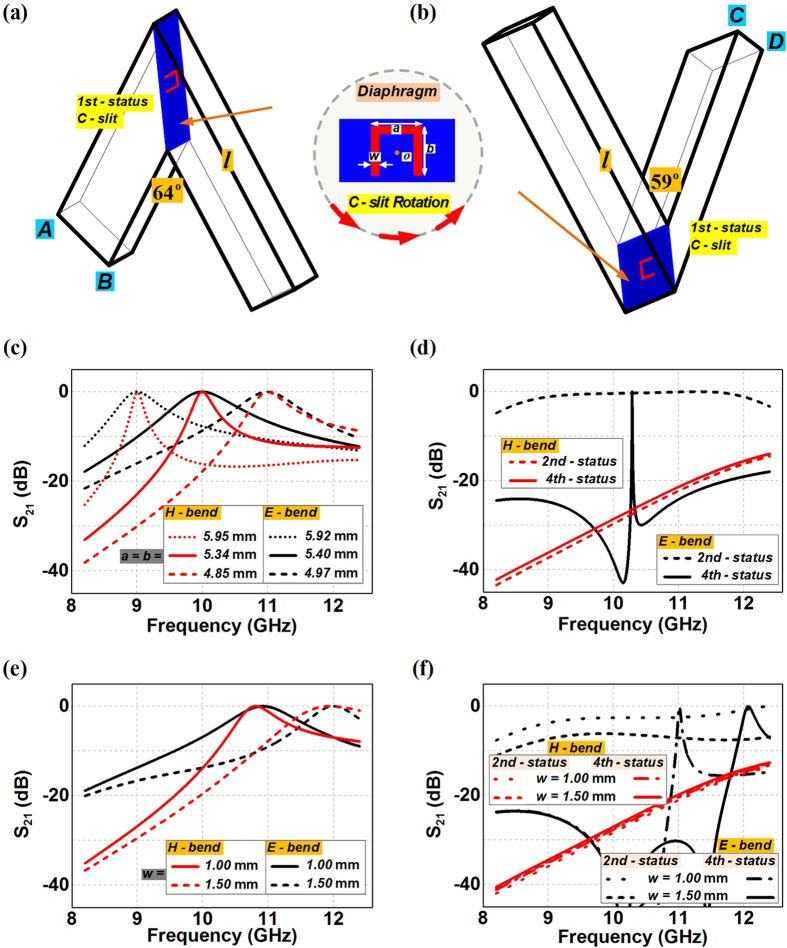
Configurations of the sharp rectangular waveguide bends with C-slit diaphragms and their transmission properties. *o* refers to the center of the C-slit diaphragms. (**a**) C-slit diaphragm based H-bend. (**b**) C-slit diaphragm based E-bend. (**c**) The transmissions from the C-slit diaphragm based waveguide bends having the 1st- or the 3rd-status C-slit with different side lengths. (**d**) The transmissions from the C-slit diaphragm based waveguide bends having the 2nd- and the 4th-status C-slit. (**e**) The transmissions from the waveguide bends having the 1st- or the 3rd-status C-slit with different slit widths. (**f**) The transmissions from the C-slit diaphragm based waveguide bends having 2nd- and the 4th-status C-slit with different slit widths. In (**c**) and (**d**), *w* = 0.5 mm. In (**d**,**e** and **f**), *a* = *b* = 5.34 mm in the H-bend and *a* = *b* = 5.40 mm in the E-bend.

**Figure 3:**
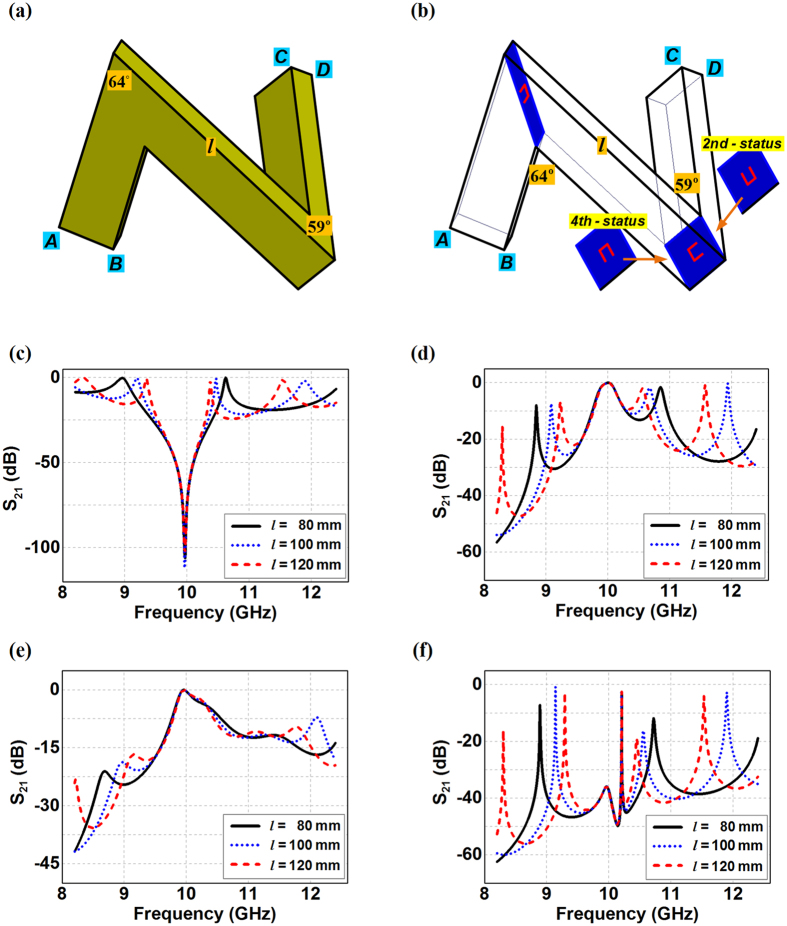
Configurations of the 3D cascade sharp waveguide bend with(out) the C-slit diaphragms and their transmission properties. (**a**) Bare 3D cascade sharp waveguide bend. (**b**) 3D cascade waveguide bend with C-slit diaphragms. (**c**) The transmissions from the 3D cascade sharp waveguide bend without C-slit diaphragms. (**d**) The transmissions from the 3D cascade sharp waveguide bend with diaphragms having both the 1st-status C-slits in the H-bend and the E-bend. (**e**) The transmissions from the 3D cascade sharp waveguide bend with diaphragms having the 1st-status C-slit in the H-bend and 2nd-status C-slit in the E-bend. (**f**) The transmissions from the 3D cascade sharp waveguide bend with diaphragms having the 1st-status C-slit in the H-bend and 4th-status C-slit in the E-bend. In (**c**–**f**), the length of the transit waveguide segment varies from 80 mm to 120 mm.

**Figure 4:**
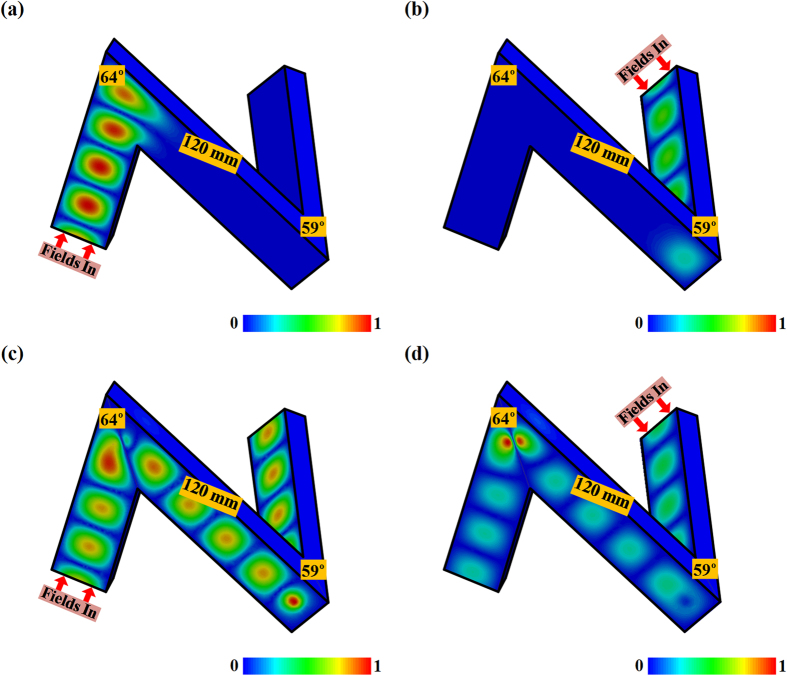
E-field distributions through the 3D cascade sharp waveguide bend with(out) the C-slit diaphragms at 10 GHz. (**a**) Bare 3D cascade sharp waveguide bend fed from the H-bend. (**b**) Bare 3D cascade sharp waveguide bend fed from the E-bend. (**c**) 3D cascade sharp waveguide bend with C-slit diaphragms fed from the H-bend. (**d**) 3D cascade sharp waveguide bend with C-slit diaphragms fed from the E-bend. The E-fields are normalized by 5.14 × 10^3^ V/m (**a**), 8.77 × 10^3^ V/m (**b**), 3.32 × 10^3^ V/m (**c**), 9.17 × 10^3^ V/m (**d**), respectively. In (**c**) and (**d**), the diaphragms have the 1st-status C-slit in the H-bend and 2nd-status C-slit in the E-bend.

**Figure 5:**
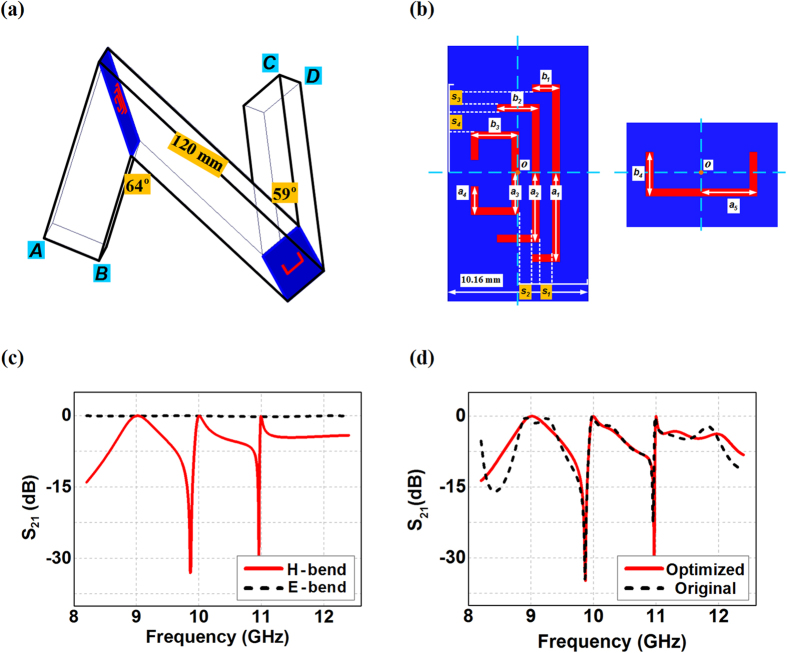
Multi-band transmission designs of the 3D cascade sharp waveguide bend. (**a**) Configurations of the multi-band 3D cascade sharp waveguide bend with diaphragms having the nested C-slits and the optimized 2nd-status C-slit. (**b**) Magnified pictures of the C-slits in (**a**) for the multiband and wideband transmission designs. The structural parameters of the nested C-slits are *a*_1_ = 6.4 mm, *a*_2_ = 5 mm, *a*_3_ = 3 mm, *a*_4_ = 2 mm, *b*_1_ = 2 mm, *b*_2_ = 3.05 mm, *b*_3_ = 3.43 mm, *s*_1_ = 1 mm, *s*_2_ = 1 mm, *s*_3_ = 0.9 mm, and *s*_4_ = 1.5 mm. The structural parameters of the optimized 2nd-status C-slit are *a*_5_ = 5 mm, and *b*_4_ = 4 mm. The slit widths are all kept as 0.5 mm. *o* refers to the center of the C-slit diaphragms. (**c**) Transmission properties of the single H-bend with multi-band C-slit diaphragm and the single E-bend with wideband C-slit diaphragm. (**d**) Transmission properties of multi-band 3D cascade sharp waveguide bend. The original transmission refers to the multi-band 3D cascade sharp waveguide bend with the wideband diaphragm having the original 2nd-status C-slit in Fig. 2(b).

**Figure 6:**
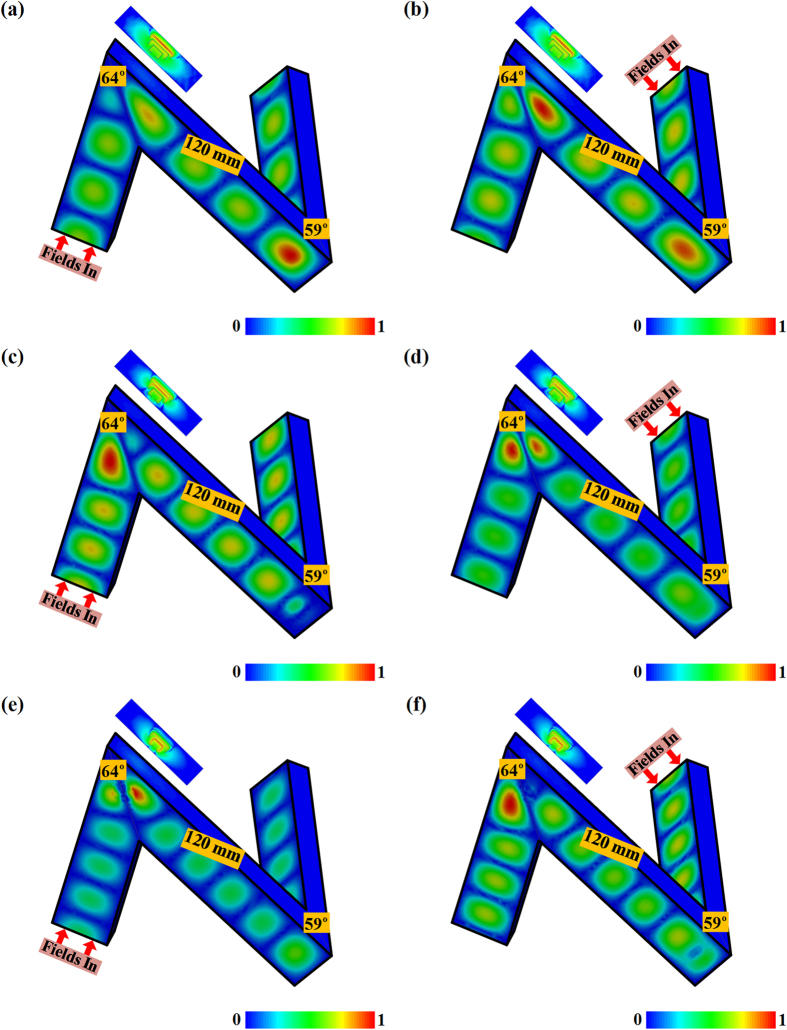
E-field distributions through the multiple band 3D cascade sharp waveguide bend. (**a**) Fed from the H-bend at 9 GHz. (**b**) Fed from the E-bend at 9 GHz. (**c**) Fed from the H-bend at10 GHz. (**d**) Fed from the E-bend at 10 GHz. (**e**) Fed from the H-bend at 11 GHz. (**f**) Fed from the E-bend at 11 GHz. The E-fields are normalized by 4.36 × 10^3^ V/m (**a**), 4.17 × 10^3^ V/m (**b**), 3.76 × 10^3^ V/m (**c**), 5.26 × 10^3^ V/m (**d**), 7.20 × 10^3^ V/m (**e**), and 4.07 × 10^3^ V/m (**f**), respectively. The inset pictures refer to the E-field distributions of the multiband C-slit diaphragm at different frequencies when the TE_10_ mode gets through. The E-fields are normalized by 1.78 × 10^5^ V/m (**a**), 3.07 × 10^5^ V/m (**b**), 4.69 × 10^5^ V/m (**c**), 1.17 × 10^6^ V/m (**d**) 2.67 × 10^3^ V/m (**e**), and 8.97 × 10^5^ V/m (**f**), respectively.

**Figure 7:**
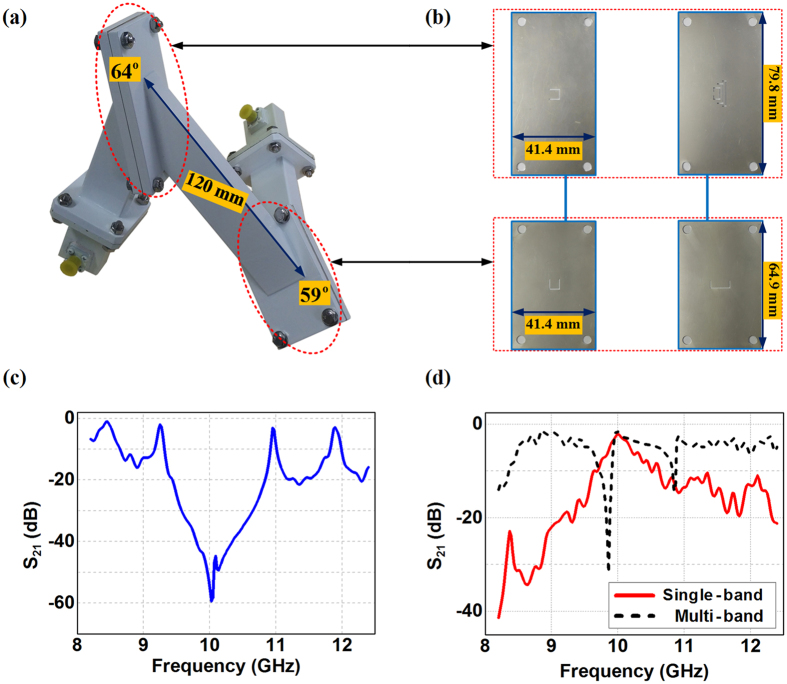
Experiments of the 3D cascade waveguide bends based on C-slit diaphragms. (**a**) Manufactured photo of the 3D cascade waveguide bends with WR90 waveguide feed. (**b**) Manufactured photo of the C-slit diaphragms. (**c**) Measured results of the transmission coefficients of the bare 3D cascade waveguide bends. (**d**) Measured results of the transmission coefficients of the single band and multi-band 3D cascade waveguide bends with C-slit diaphragms.
